# Supplementary material for: The role of critical care nurses in shared decision-making for patients with severe heart failure: A qualitative study
Source: PLoS One. 2023 Jul 20;18(7):e0288978. doi: 10.1371/journal.pone.0288978 (PMC10358911; doi:10.1371/journal.pone.0288978)
Supplement: S1 Table — CCNS, certified nurse specialist(s) in critical care nursing; CRRT, continuous renal replacement therapy; ECMO, extracorporeal membrane oxygenation; HTx, heart transplantation; ICU, intensive care unit; VAD, ventricular assist device. (DOCX) [file pone.0288978.s001.docx]

**S1 Table. Additional quotes about patients’ difficult situations in decision-making.**

| **Theme** | **Exemplifying quote** |
| --- | --- |
| **Painful decisions under uncertainties** | (At the husband’s insistence, the patient began receiving the best possible treatment (such as ECMO, etc.), but there were no signs of improvement.) Furthermore, when the possibility that the patient’s brain might be diseased came to light, the patient’s husband said, “I can’t bear the sight (of the patient receiving treatment).” He requested to stop (the treatment), as he wanted to “bring her home.” (CCNS: A) |
|  | (CRRT may be poorly indicated for patients with end-stage heart failure). In some cases, the physician would take a chance and start CRRT, which could lead to complications and eventual death. Therefore, considering the pros and cons, CRRT is not presented (to the patient as an option) in some cases. (CCNS: C) |
|  | (The patient’s condition deteriorated again after staying in the ICU for 4 months and having weaned off ECMO and mechanical ventilation). The patient went into shock immediately. Thus, the family was forced to decide on the treatment once again, even though they had (previously) decided not to use the machine (for mechanical ventilation) anymore (even if the condition worsened in the future). (CCNS: E) |
| **Tense relationships** | The patient (who had not been informed of his condition) became increasingly frustrated. He started refusing care from the nurses and verbally abusing his wife who was staying in his hospital room. I talked to his wife and asked her why they had not told the patient about his condition. His wife said that they were “afraid that he would get discouraged.” (CCNS: D) |
|  | (The patient himself understood his situation of being in and out of the hospital for heart failure.) The patient was aware that he must go through painful treatment each time; thus, he said that he had “no regrets even if my life is about to end, but my family does not understand this.” This implies that he was troubled, as he could not convey his feeling to his family. (CCNS: H) |
| **Wavering emotions during decision-making** | To the family, an acute exacerbation of the patient’s disease was unexpected. Once the patient becomes unconscious, the family members are left make the decisions. (Even if the acute exacerbation is explained to the family), they can become aggressive when they do not fully understand the explanation they received before surgery or admission. (CCNS: B) |
|  | The patient went into shock (immediately after the recurrence of the infection). It had been decided to not use the machine (for mechanical ventilation), but the patient seemed to be in pain and dyspnea. When the doctor told the family that the patient might die within 3 h if nothing was done, they immediately expressed that they would not want that to happen. (CCNS: E) |
|  | The patient with heart failure had been asking his family to ease his painful situation by not prolonging his life. When the heart deteriorated, the patient’s sons, in desperation, changed their minds about intubating the patient (against the patient’s wish). (CCNS: F) |
| **Difficulties in coping with worsening medical conditions** | Morphine was administered to ease the patient’s pain. The patient had to decide whether to accept the morphine for his breathing difficulties, (however the use of morphine could also end his life). The patient was tired and not at capacity to make decisions. He had difficulties deciding for himself. (CCNS: B) |
|  | They were informed of the pros (of the VAD and HTx). I would tell them the cons, which would give the patients a bad impression. In this situation, the patient did not want to hear any unpleasant news while in distress with advanced heart failure. As such, before I came in, the patient was talking to the nurses and laughing, but when I arrived, their faces took on a stern expression. (CCNS: J) |
| **Patients’ wishes that are difficult to realize or estimate** | I was told that the unconscious patient had expressed repeatedly over several decades (even before he fell ill), that he was ready to die. In reality, even his family did not understand why the patient was saying this. Since we did not know the patient’s true feelings, we could not be certain that his words meant that he did not want his life to be saved. (CCNS: E) |
|  | The patient was unconscious. The patient was living with his mother who was in her late 80s. He did not have siblings; thus, we were unsure of what to do (who to go to for a proxy decision to accept or decline VAD surgery). His mother, being an older adult, was slightly mentally unstable and unable to respond to the explanation. (CCNS: H) |
| **Difficulties in transitioning from advanced medical care** | (I asked the deputy head nurse what she thought about readmissions of patients with end-stage heart failure in home care), she said that this (readmitting a patient because of difficulties with home care) was the role of hospitals “in this day and age.” She also said that she “does not understand why our hospital does not provide palliative care to those patients.” (CCNS: E) |
|  | Even if we determine that the patient can be discharged from the hospital after VAD implantation, the patient may not actually be discharged until a month later. The patient may be in a condition where they could be discharged, but we may be unable to do so. This is a problem concerning the patient’s courage and the family’s acceptance. What should we do to move forward? (CCNS: J) |

CCNS, certified nurse specialist in critical care nursing; CRRT, continuous renal replacement therapy; ECMO, extracorporeal membrane oxygenation; HTx, heart transplantation; ICU, intensive care unit; VAD, ventricular assist device.
