# Supplementary material for: The role of critical care nurses in shared decision-making for patients with severe heart failure: A qualitative study
Source: PLoS One. 2023 Jul 20;18(7):e0288978. doi: 10.1371/journal.pone.0288978 (PMC10358911; doi:10.1371/journal.pone.0288978)
Supplement: S2 Table — CCNS, certified nurse specialist(s) in critical care nursing; HTx, heart transplantation; ICU, intensive care unit; VAD, ventricular assist device. (DOCX) [file pone.0288978.s002.docx]

**S2 Table. Additional quotes regarding the roles of critical care nurses in shared decision-making.**

| **Theme** | **Exemplifying quote** |
| --- | --- |
| **The search for meaning and values** | In a near-end-of-life situation, the patient was unconscious but his blood pressure was still maintained. I talked to his wife in his hospital room and asked about the kind of person he was, and I heard stories about the hardships the couple had faced together. Eventually, we were both smiling. By listening and responding to their stories, I tried to alleviate the wife’s grief. (CCNS: C) |
|  | (I sometimes ask patients who have been saved), “Now that you’ve been saved, what do you value in life (going forward) that makes life meaningful ?” or “What do you want to value in life?” (A dialogue between a patient and a nurse is) not about whether to live or die, or to be intubated or not, or about treatment choices. Instead, we should be able to talk (about values on a regular basis). (CCNS: F) |
|  | The new battle begins, (not just when deciding whether to go for a VAD implantation and HTx or not), but when complications and sequelae occur after a VAD implantation, when the patient is truly lost again and the family actually takes an active role in decision-making. Ideally, I think the patients and their family should understand, thus, I try to talk about specifics. (CCNS: J) |
| **Partnership** | I believe that everything starts with the sharing of values and the formation of trust. For example, if we are in a situation where we can grieve with the family, or laugh a little, or share memories together. (If we share those feelings related to decision-making with the patient/family), then I think we should also share their feelings relating to the outcome of the chosen treatment. (CCNS: C) |
|  | It was as if (in reality) the surgeon decided (the treatment policy), but (formally) the wife agreed to operate on behalf of the patient. It changed her husband's life so much. I was concerned about her anxiety and the outlook she might have. I carefully observed how the wife was spending this time (being uncertain about the future). (CCNS: E) |
|  | (Regarding the intubation of a patient who suddenly deteriorated) There was a strong feeling from the family that they wanted to honor what the patient had been saying all along (she did not want to be intubated). While acknowledging that point, I tried to convey the views of the healthcare professionals and gradually gain the understanding of the families. (CCNS: F) |
| **Rights advocacy** | In the ICU, the patient was sometimes not informed, but the family would make a surrogate decision (of no further treatment). The patient was in pain and asked, "What is going to happen to me? I do not think I will make it.” The ICU nurses sincerely responded by saying, “I understand how you feel.” Even if the family does not want to inform the patient, the patient can still sense it. (CCNS: F) |
|  | (Even if I have doubts about the treatment plan as a nurse), I think that it would be easier if I keep quiet and listen. If I see, hear, and say something, I will not be able to back out. I struggle with that but I think that is what makes me a professional. There are some painful aspects, but mostly, I think it is precious. (CCNS: H) |
|  | (The patient’s family wanted me to tell them if the chance of recovery was slim). I asked the surgeon for an explanation. He said, “There is still a chance that the patient may be cured. I have not given up on treatment. What are you talking about?” As for whether I could have asked for someone’s help and discussed this further with the surgeon, I think this is a challenge for the future. (CCNS: I) |
| **Situation assessment** | We held a multidisciplinary conference on this subject (thoughts of families who are hesitant about continuing treatment). I was concerned about how to create an environment conducive to discussions at the conference. As a CCNS, I tend to be a little strategic, so I guide the conference so that it would be easier for multiple healthcare professionals to speak. (CCNS: A) |
|  | At least, I wondered what I could do in the current situation. I flipped through all the patient’s medical records. It had been 10 years since he developed the disease, and the patient had a long treatment history. (He is now over the age for HTx, but he had considered HTx before.) I started by going through his medical record to see what he valued. (CCNS: C) |
|  | The patient was depressed, thinking that she would not ever recover. But now that her condition was improving, I thought that surgery may be possible. At that time, I asked the cardiac surgeon about the possibility of having surgery, and he said that while it was quite difficult, there was a chance that it could be done. (CCNS: F) |
| **Mediation** | The (patient’s) sense of value was, “If the doctor thinks it is treatable, I want to be treated.” However, the doctor’s explanation was inadequate (I explained the possibility of treatment). I would not say anything that is inconsistent with what the doctor would say. If there is anything I do not understand, I would always provide further information only after asking the doctor. (CCNS: C) |
|  | I (as a member of the palliative care team) am not directly involved with the patients. Thus, there are times when I cannot establish rapport with them. Therefore, I try to follow the process of sharing the intentions of the psychologists and psychiatrists with the nurses in charge and helping them make decisions together while also being involved. (CCNS: I) |
|  | I sometimes coordinate patient-to-patient conversations so that pre-operative patients can hear from those who have had a VAD. These patients see others dying around them and know that they will be readmitted to the hospital. The patients also know that their families, for example, are supporting them in their treatment. I tell these patients, “Please talk frankly about such things.” (CCNS: J) |
| **Co-creation** | (Since the patient was at risk of sudden death,) I told the patient that “We will talk about how to die without denying it, because that day may actually come. But we will also talk about how to live until then.” (I had been caring for this patient for a long time,) I did this while providing feedback to the ward nurses (so that his wishes, such as staying out overnight, can be fulfilled). (CCNS: D) |
|  | (Even if the attending physician is not considering home care,) if the patient and their family really, really strongly desire to go home, I thought it would be okay to try to have a positive multidisciplinary conference to see if we can make that wish come true. The family doctor might say, “I can (offer home management of inotropes)”. (CCNS: E) |
|  | I talked to the hospital directors, “We need to find a hospital for palliative care of patients with terminal heart failure and be clear about the preparations needed for the patient to be accepted.” If we do not create a palliative care model, we would be cornered. Therefore, everyone was prepared and worked on it, and we were able to pave the way. (CCNS: G) |

CCNS, certified nurse specialist in critical care nursing; HTx, heart transplantation; ICU, intensive care unit; VAD, ventricular assist device.
