# Supplementary material for: The role of critical care nurses in shared decision-making for patients with severe heart failure: A qualitative study
Source: PLoS One. 2023 Jul 20;18(7):e0288978. doi: 10.1371/journal.pone.0288978 (PMC10358911; doi:10.1371/journal.pone.0288978)
Supplement: Attachment — Submitted filename: Response_to_Reviewers.docx [file pone.0288978.s003.docx]

**Response to the reviewer comment**

We deeply appreciate the constructive comment from reviewer #1. In response to your comment, we have further revised the manuscript for greater clarity. Our response is provided below.

**Comment#1**: The conclusions provided in abstract differ from the ones provided in manuscript. It might be clearer for readers if the abstract is revised.

**Author’s response #1:**

Thank you for this suggestion. We have revised the conclusions in the abstract to ensure that they are consistent with the conclusion in the main text. We have also revised the aim in the abstract to accommodate the word limit.

Page 2, lines 42-43 and Page 3, lines 44-45

**Conclusions**: Patients with severe heart failure have difficulty participating in shared decision-making. Critical care nurses should collaborate within the nursing team to improve interprofessional shared decision-making by providing decisional support to patients that focuses on values and meaning.

Page 2, lines 25-27

“This study aimed to clarify the perceptions of critical care nurses regarding situations with patients with severe heart failure that require difficult decision-making, and their role in supporting these patients.”

Again, thank you very much for taking the time to review our paper.

Sincerely,

Noriko Inagaki

E-mail: noriko.inagaki@nrs.setsunan.ac.jp
